# The safety and efficacy of radiofrequency ablation in benign pediatric thyroid disease in the US: An initial case series

**DOI:** 10.1002/lio2.1198

**Published:** 2024-01-10

**Authors:** Grace S. Kim, Hilary Seeley, Julia Noel, Iram Ahmad, Kara Meister

**Affiliations:** ^1^ Children's Thyroid Clinic at Stanford Children's Health Stanford California USA; ^2^ Division of Pediatric Otolaryngology, Department of Otolaryngology–Head & Neck Surgery Stanford University School of Medicine Palo Alto California USA; ^3^ Division of Pediatric Endocrinology, Department of Pediatrics Stanford University School of Medicine Palo Alto California USA; ^4^ Santa Clara Valley Medical Center, Department of Otolaryngology–Head & Neck Surgery Stanford University School of Medicine Palo Alto California USA

**Keywords:** lingual thyroid, pediatric thyroid disease, radiofrequency ablation, thyroid nodule

## Abstract

**Objective:**

To evaluate the efficacy and safety of radiofrequency ablation (RFA) for benign nonfunctional thyroid nodules or functional lingual thyroid gland in a pediatric population.

**Methods:**

Four pediatric patients (four female; mean age 13.50 ± 4.04, range 8–17 years) with either benign thyroid nodules or mildly obstructive lingual thyroid glands were treated with RFA from 2020 to 2021 were evaluated. The inclusion criteria for RFA therapy were (i) age < 18 years; (ii) benign cytopathological results on ultrasound guided fine needle aspiration; (iii) pressure or pain symptoms caused by the thyroid nodules; (iv) dysphagia or obstruction caused by the lingual thyroid tissue; (v) follow up for >6 months with otolaryngology or endocrinology.

**Results:**

Two patients had benign non‐functioning thyroid nodules and two had mildly obstructive functioning lingual thyroid glands. Mean follow up was 10.75 ± 4.79 months. Each patient underwent one RFA session with no complications. For the patients with thyroid nodules, there was >74% reduction in nodule size at last follow up with improvement in neck swelling and pain. For the patients with lingual thyroid glands, both did not have any other functional thyroid gland identified. Both had visible decrease in size of the gland as visualized transorally with improvement in dysphagia and obstructive symptoms when lying flat.

**Conclusion:**

RFA is a safe and effective option for managing benign thyroid nodules and lingual thyroid glands in a pediatric patient population.

**Level of evidence:**

4.

## Graphical abstract

This is a description of the safety and feasibility of four cases using radiofrequency ablation to treat benign pediatric thyroid pathology.

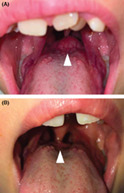

## 1 INTRODUCTION

The incidence of thyroid nodules in a pediatric population ranges from 0.2% to 5%.^1^ Even rarer is ectopic lingual thyroid tissue, reported to have an overall prevalence of 1 in 100,000 patients.^2^ Although most lingual thyroid tissue and thyroid nodules are benign in pediatric patients, they can grow in size and cause compressive symptoms such as neck pain/discomfort, dysphagia, or dysphonia. Surgical management can be offered in symptomatic patients, but surgery carries a number of risks, including recurrent laryngeal or superior laryngeal nerve injury, hypoparathyroidism, hypocalcemia, bleeding and scarring.^3^, ^4^ Therefore, radiofrequency ablation (RFA) is emerging^5^ as an alternative treatment option for patients with benign disease. While the technique, safety, and outcomes have been studied more extensively in adult patients, there is limited data on its use in pediatric patients.

Due to the gap in knowledge, our objective was to evaluate the efficacy and safety of RFA for benign, nonfunctional thyroid nodules or functional lingual thyroid tissue in a pediatric population.

## 2 MATERIALS AND METHODS

A retrospective review of pediatric patients undergoing RFA at a tertiary pediatric hospital by pediatric otolaryngologists over a one‐year period from 2020 to 2021 was performed. This study was approved by our Institutional Review Board.

The inclusion criteria for RFA therapy were (i) age < 18 years; (ii) two documented benign cytopathological results on ultrasound‐guided fine needle aspiration of thyroid nodules; (iii) pressure or pain symptoms caused by the thyroid nodules; (iv) dysphagia or obstruction caused by the lingual thyroid tissue; (v) follow up for >6 months with otolaryngology or endocrinology. Volume reduction of the nodule or lingual thyroid after the procedure, changes in symptoms, any complications arising from the RFA and the need for post‐operative thyroid hormone supplementation was evaluated.

### 2.1 Pre‐procedural evaluation

For patients with nodules, ultrasound‐guided FNA was performed twice. Resulting pathology was benign thyroid nodule for all biopsies. Biopsies were at least 3 months apart, and at least 3 months from RFA. All patients had a formal neck ultrasound to evaluate the thyroid and all levels of the neck bilaterally and an additional surgeon‐performed ultrasound on the day of RFA, as is our institutional protocol prior to any procedural intervention for patients with thyroid pathology. The patients with nodules had single thyroid nodules and standard ultrasound dimensions were used to calculate nodule size. Nonetheless, the disclosure that RFA could miss or obscure an underlying thyroid malignancy was extensively discussed with the family prior to signing informed consent.

### 2.2 Surgical technique

All RFA procedures were performed under general anesthesia. For the patients with thyroid nodules, intraoperative ultrasound was performed to locate the target nodules. One of the patients with a thyroid nodule was intubated with a NIM tube (Medtronic); the other patient's airway was managed with an LMA. Local anesthesia was administered. Saline was injected around the nodules to serves a thermal buffer given the heat generated by the RFA. Using ultrasound guidance, the ablation of the thyroid nodule was completed with a RF generator and 18‐gauge electrode set at a power of 20–45 W.

RFA of lingual thyroid tissue was performed with a Celon wand. The patients were positioned with a Jennings mouth gag and a Friedman rake was used to augment exposure. This allowed direct visualization of the lingual thyroid tissue. The Celon wand was inserted and used to ablate the tissue at a setting of 18. Total wattage recording was not available on the device.

## 3 RESULTS

Four female pediatric patients were included in the study, ranging in age from 8 to 17 years old when they underwent RFA. The mean age was 13.50 ± 4.04 years. Two patients had benign non‐functioning thyroid nodules and two had mildly obstructive functioning lingual thyroid tissue. Mean follow up was 16.75 ± 4.79 months. Each patient underwent one RFA session with no complications.

**FIGURE 1:**
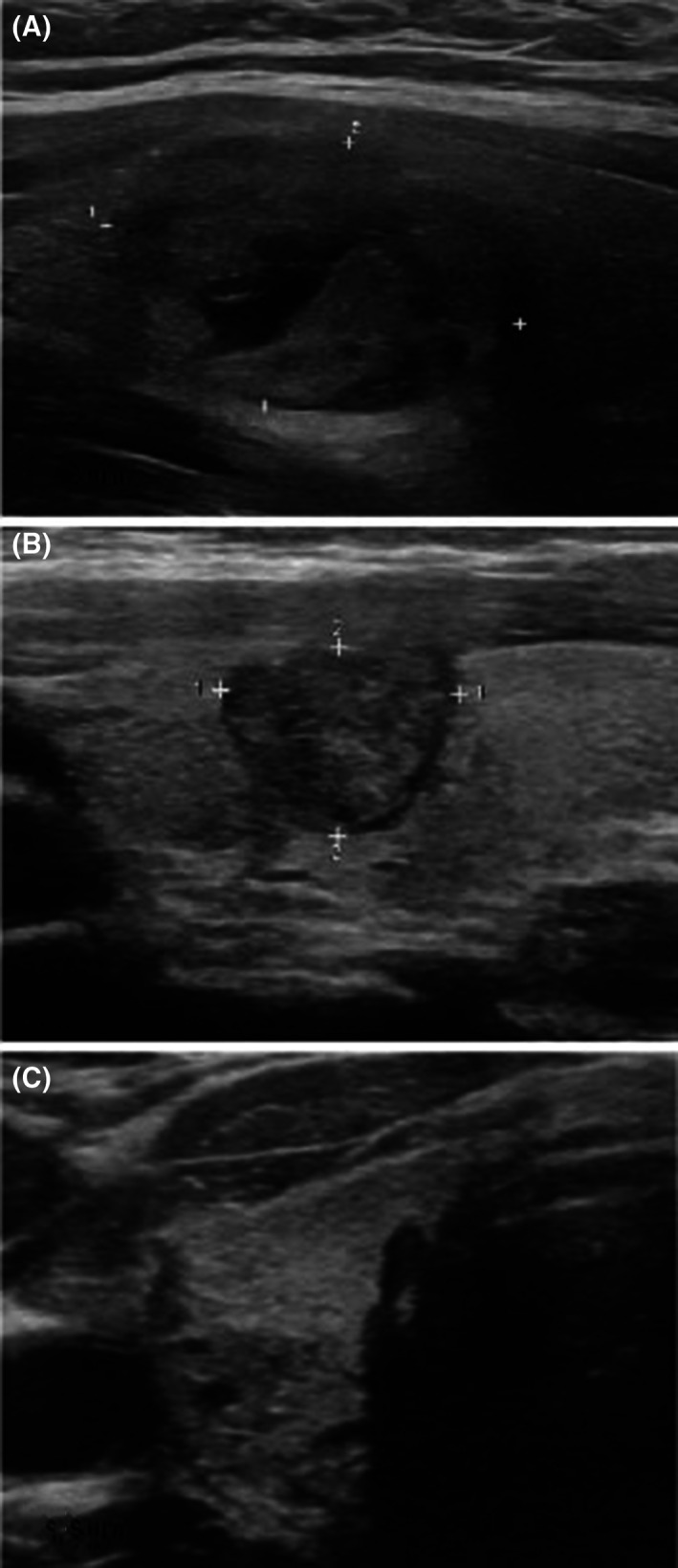
Preoperative (A) ultrasound of a thyroid nodule compared with a postoperative (B) ultrasound at 3 months after RFA for one patient, showing decrease in size. In contrast, postoperative ultrasound 2 years after RFA (C) for another patient that shows loss of a distinct nodule border.

For the patients with thyroid nodules, they underwent preprocedural and postprocedural thyroid ultrasounds. Both patients had nodules initially described as mixed solid/cystic. One patient had a 3.8 cm^3^ right midpole nodule that decreased to 0.8 cm^3^ on most recent ultrasound, representing a 79% volume reduction (Figure 1A,B). The other patient had a 3.4 cm^3^ right lobe nodule, which was measured as 5.0 cm^3^ on most recent imaging, but this nodule showed an indistinct border and more homogenous appearance on ultrasound, no persistent cystic component, and was no longer clinically palpable by the medical team or patient (Figure 1C). This patient was diagnosed with autoimmune thyroiditis with elevated anti‐thyroglobulin antibodies but normal thyroid function tests after the ablation. Neither patient with thyroid nodules who underwent RFA required exogenous thyroid hormone after the procedure.

**FIGURE 2:**
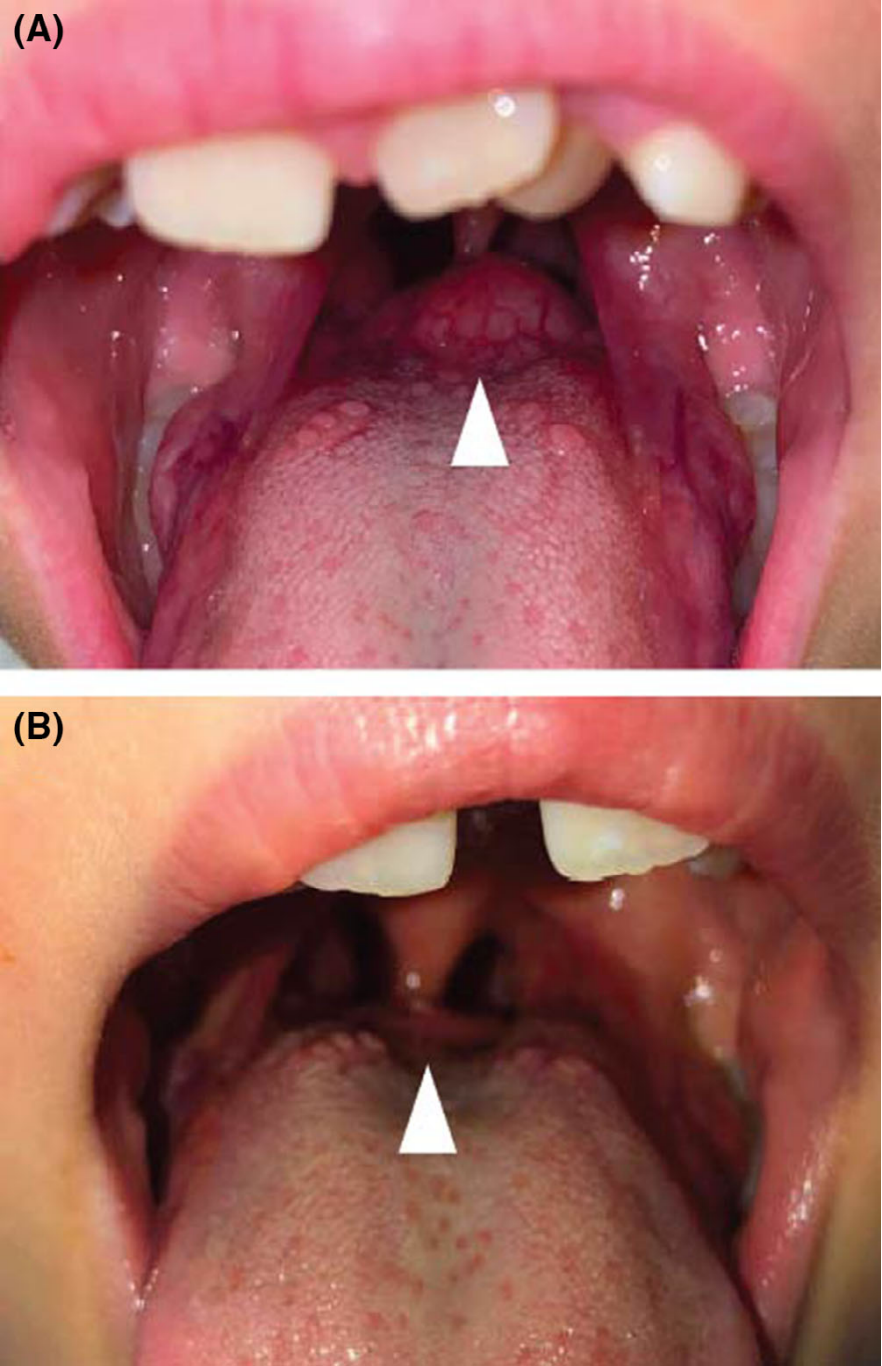
Preoperative (A) appearance of a lingual thyroid after treatment with levothyroxine for 12 months versus postoperative (B) appearance at 4 weeks after RFA.

Both patients with lingual thyroid glands did not have any other functional thyroid gland identified on imaging. Both patients were started on thyroid hormone to both shrink the lingual thyroid tissue and treat their hypothyroidism with a goal of normal TSH. Both had failed medical management defined as persistent dysphagia despite at least 12 months of thyroid hormone supplementation. Both had visible decrease in size of the gland as visualized transorally with improvement in dysphagia and obstructive symptoms when lying flat (Figure 2). After RFA, both continued to receive exogenous thyroid hormone supplementation.

## 4 DISCUSSION

Minimally invasive techniques to address both benign and malignant thyroid pathology have been previously reported, primarily in an adult patient population. Available techniques include chemical ablation (e.g., tetracycline or ethanol) and energy‐based ablation (e.g., laser, radiofrequency, microwave or ultrasound). He and colleagues found that RFA was superior to other ultrasound‐guided percutaneous ablation techniques for reducing benign thyroid nodules in both short‐ and long‐term follow up. Subgroup analysis showed that while RFA thermal ablation was superior in volume decrease for solid or predominantly solid benign nodules, chemical ablation with ethanol was more efficient for cystic nodules.

As ultrasound‐guided thermal ablations have become more mainstream for treating benign thyroid nodules in adults, we have more data on safety and efficacy. A recent meta‐analysis showed that with long term follow up of at least 36 months, the complication rate was an acceptable 3.8%.^6^ Although there has been favorable efficacy in volume reduction reported for benign nodules, longer periods of follow up are needed to clarify the potential regrowth rate.^7^, ^8^, ^9^, ^10^, ^11^ One impact on the risk of regrowth is not only the composition of the original nodule but also the ablation method and completion of margin ablation.^12^ Due to the increased maneuverability and moving‐shot technique of RFA, the electrode can more easily be moved to target marginal vessels, leading to improved margin ablation.^13^ This is different from the laser whose electrode is placed at the center of the nodule during ablation, limiting ablation of the nodule margin.^9^ Our study highlights that nodule size alone may not be the sole metric of successful treatment, but changes in margin appearance and nodule composition without further growth may also indicate effective treatment. Again, longer term data will help elucidate effective treatment.

Thyroid nodules are less common in children but carry a higher risk of malignancy. Therefore, the criteria for obtaining an FNA, the role of molecular data with indeterminate FNA results, and the treatment options currently available for pediatric patients differ from those for adult patients.^14^ There is limited data looking at the safety and efficacy of non‐surgical treatment options in pediatric patients. One retrospective study looked at 62 pediatric patients with 70 benign thyroid nodules treated with RFA followed over 4 years.^15^ They noted decrease in volume was greatest at 1 year but 22.9% of nodules had regrowth, over half (66.7%) of which had greater volume than their initial presentation. Some underwent repeat ablation with limited repeat efficacy. Bilateral nodules, low vascularity and low cystic components were risk factors correlating with decreased efficacy. Hong et al showed similarly that RFA was a safe technique for pediatric benign thyroid disease with good volume reduction and improved compressive symptoms with an average of two RFA sessions when followed for over 36 months.^16^ Because RFA is relatively new to the US healthcare system, there have been no publications on the US pediatric population to date.

The data on RFA use in lingual thyroid tissue is very limited with isolated case reports of use in an adult^17^ and a child.^18^ Such level of data in the literature presents limited standardization of techniques and insight into generalizable outcomes. Our case series used an impedance‐controlled ablation technique for all four patients. This allowed for minimal disruption of the mucosa and overlying surface while administering controlled ablation of the deeper components. This was particularly important in terms of surgical safety for one of our patients who was a Jehovah's witness and declined blood products. Although a small case series, we are able to show feasibility a safety of RFA for treating lingual thyroid tissue as an alternative to thyroid hormone therapy to reduce the size or surgical excision.

There are several limitations to our case series. The most significant is the small patient number and limited follow up. The current experience is too limited to make conclusions as to whether RFA is a correct and safe treatment in pediatric thyroid nodules from a long‐term perspective. One patient in our experience did have a change of the ultrasound appearance and physical exam characteristics of the nodule which could make long term surveillance more difficult. This is significant given the higher likelihood of an underlying malignancy in pediatric thyroid nodules versus the adult population. Future studies and directions should include prospective series with standardized patient‐reported outcomes measures (PROMs), development of pediatric‐focused PROMs for thyroid interventions, and long‐term follow up.

Limited data exists on the feasibility of chemical or thermal ablative techniques for malignant thyroid disease. The use of laser ablation for primary papillary microcarcinoma has shown either disappearance of the lesion on surveillance imaging or decrease in size of the lesion.^19^, ^20^ Excision of the lesion after laser ablation has also shown that the lesions lack viability as demonstrated on immunohistochemical analysis of the surgical specimens.^21^ The use of such minimally invasive techniques of ablation have also been studied in recurrent malignant thyroid disease which showed reduction in size and similar recurrence rates (<8%) as with surgery.^22^, ^23^, ^24^ Therefore, chemical and thermal ablation techniques may serve as an alternative treatment option for those who are not candidates for surgical resection. The authors caution, however, against the use of RFA for malignant disease in the pediatric population because the disease has a significant risk of multifocality, likely due to underlying genetic predispositions which are distinctly different than those in the adult population. Until the molecular landscape of pediatric thyroid cancer can be better elucidated to aid in patient selection for focal treatment, RFA is not likely to be recommended as a first‐line treatment for malignant thyroid pathology in children.

## 5 CONCLUSION

The limited data highlights our gap in knowledge regarding minimally invasive pediatric thyroid care. As we better understand pediatric thyroid disease and the ways in which they differ from adult thyroid pathology, our ability to offer non‐surgical treatment options for pediatric patients may continue to develop.

## CONFLICT OF INTEREST STATEMENT

The authors declare no conflict of interest.

## References

[1] Divarci E , Celtik U , Dökümcü Z , et al. Management of childhood thyroid nodules: surgical and endocrinological findings in a large group of cases. J Clin Res Pediatr Endocrinol. 2017;9(3):222‐228. 28387647 10.4274/jcrpe.4272 PMC5596803

[2] Anand SS , Sood V , Kumar PG , Suryanarayna KM , Kotwal N . Lingual thyroid. Med J Armed Forces India. 2006;62(2):184‐185. 27407890 10.1016/S0377-1237(06)80068-7 PMC4921966

[3] Hanba C , Svider PF , Siegel B , et al. Pediatric thyroidectomy. Otolaryngol Head Neck Surg. 2017;156:360‐367. 28145836 10.1177/0194599816677527

[4] Baumgarten HD , Bauer AJ , Isaza A , Mostoufi‐Moab S , Kazahaya K , Adzick NS . Surgical management of pediatric thyroid disease: complication rates after thyroidectomy at the Children's Hospital of Philadelphia high‐volume pediatric thyroid center. J Pediatr Surg. 2019;54:1969‐1975. 30902456 10.1016/j.jpedsurg.2019.02.009

[5] He L , Zhao W , Xia Z , Su A , Li Z , Zhu J . Comparative efficacy of different ultrasound‐guided ablation for the treatment of benign thyroid nodules: systematic review and network meta‐analysis of randomized controlled trials. PLoS One. 2021;16(1):e0243864. 33471820 10.1371/journal.pone.0243864 PMC7816973

[6] Cho SJ , Baek JH , Chung SR , Choi YJ , Lee JH . Long‐term results of thermal ablation of benign thyroid nodules: a systematic review and meta‐analysis. Endocrinol Metab. 2020;35:339‐350. 10.3803/EnM.2020.35.2.339 PMC7386110 32615718

[7] Magri F , Chytiris S , Molteni M , et al. Laser photocoagulation therapy for thyroid nodules: long‐term outcome and predictors of efficacy. J Endocrinol Invest. 2020;43:95‐100. 31321758 10.1007/s40618-019-01085-8

[8] Papini E , Rago T , Gambelunghe G , et al. Long‐term efficacy of ultrasound‐guided laser ablation for benign solid thyroid nodules: results of a three‐year multicenter prospective randomized trial. J Clin Endocrinol Metab. 2014;99:3653‐3659. 25050903 10.1210/jc.2014-1826

[9] Valcavi R , Riganti F , Bertani A , Formisano D , Pacella CM . Percutaneous laser ablation of cold benign thyroid nodules: a 3‐year follow‐up study in 122 patients. Thyroid. 2010;20:1253‐1261. 20929405 10.1089/thy.2010.0189

[10] Lim HK , Lee JH , Ha EJ , Sung JY , Kim JK , Baek JH . Radiofrequency ablation of benign non‐functioning thyroid nodules: 4‐year follow‐up results for 111 patients. Eur Radiol. 2013;23:1044‐1049. 23096937 10.1007/s00330-012-2671-3

[11] Sim JS , Baek JH , Lee J , Cho W , Jung SI . Radiofrequency ablation of benign thyroid nodules: depicting early sign of regrowth by calculating vital volume. Int J Hyperthermia. 2017;33:905‐910. 28540795 10.1080/02656736.2017.1309083

[12] Sim JS , Baek JH . Long‐term outcomes following thermal ablation of benign thyroid nodules as an alternative to surgery: the importance of controlling regrowth. Endocrinol Metab. 2019;34:117‐123. 10.3803/EnM.2019.34.2.117 PMC6599899 31257739

[13] Baek JH , Lee JH , Valcavi R , Pacella CM , Rhim H , Na DG . Thermal ablation for benign thyroid nodules: radiofrequency and laser. Korean J Radiol. 2011;12:525‐540. 21927553 10.3348/kjr.2011.12.5.525 PMC3168793

[14] Goldfarb M , Dinauer C . Differences in the management of thyroid nodules in children and adolescents as compared to adults. Curr Opin Endocrinol Diabetes Obes. 2022;29(5):466‐473. 35777975 10.1097/MED.0000000000000754

[15] Li L , Qiu X . Safety and efficacy of ultrasound‐guided radiofrequency ablation for benign nonfunctional thyroid nodules in children: a retrospective study of 62 patients with over four years of follow up. Thyroid. 2022;32(5):525‐535. 34915754 10.1089/thy.2021.0454

[16] Hong MJ , Sung JY , Baek JH , et al. Safety and efficacy of radiofrequency ablation for nonfunctioning benign thyroid nodules in children and adolescents in 14 patients over a 10‐year period. J Vasc Interv Radiol. 2019;30(6):900‐906. 30956073 10.1016/j.jvir.2018.10.034

[17] Dasari SD , Bashetty NK , Prayaga NSM . Radiofrequency ablation of lingual thyroid. Otolaryngol Head Neck Surg. 2007;136:498‐499. 17321889 10.1016/j.otohns.2006.07.021

[18] Cunningham CL , Vilela RJ , Roy S . Radiofrequency ablation as an novel treatment for lingual thyroid. Int J Pediatr Otorhinolaryngol. 2011;75:137‐139. 21074866 10.1016/j.ijporl.2010.10.022

[19] Papini E , Guglielmi R , Gharib H , et al. Ultrasound‐guided laser ablation of incidental papillary thyroid microcarcinoma: a potential therapeutic approach in patients at surgical risk. Thyroid. 2011;21(8):917‐920. 21595556 10.1089/thy.2010.0447 PMC3148119

[20] Bernardi S , Dobrinja C , Fabris B , et al. Radiofrequency ablation compared to surgery for the treatment of benign thyroid nodules. Int J Endrocrinol. 2014;2014:934595. 10.1155/2014/934595 PMC4090443 25045352

[21] Valcavi R , Piana S , Bortolan GS , Lai R , Barbieri V , Negro R . Ultrasound‐guided percutaneous laser ablation of papillary thyroid microcarcinoma: a feasibility study on three cases with pathological and immunohistochemical evaluation. Thyroid. 2013;23(12):1578‐1582. 23978269 10.1089/thy.2013.0279

[22] Kim SY , Kim SM , Chang H , et al. Long‐term outcomes of ethanol injection therapy for locally recurrent papillary thyroid cancer. Eur Arch Otorhinolaryngol. 2017;274(9):3497‐3501. 28664330 10.1007/s00405-017-4660-2

[23] Fontenot TE , Deniwar A , Bhatia P , al‐Qurayshi Z , Randolph GW , Kandil E . Percutaneous ethanol injection vs reoperation for locally recurrent papillary thyroid cancer: a systematic review and pooled analysis. JAMA Otolaryngol Head Neck Surg. 2015;141(6):512‐518. 25928119 10.1001/jamaoto.2015.0596

[24] Kim JH , Yoo WS , Park YJ , et al. Efficacy and safety of radiofrequency ablation for treatment of locally recurrent thyroid cancers smaller than 2 cm. Radiology. 2015;276(3):909‐918. 25848897 10.1148/radiol.15140079

